# Ketamine Modulates Zic5 Expression via the Notch Signaling Pathway in Neural Crest Induction

**DOI:** 10.3389/fnmol.2018.00009

**Published:** 2018-02-07

**Authors:** Yu Shi, Jiejing Li, Chunjiang Chen, Yongwu Xia, Yanxi Li, Pan Zhang, Ying Xu, Tingyu Li, Weihui Zhou, Weihong Song

**Affiliations:** ^1^Department of Clinical Laboratory, Children’s Hospital of Chongqing Medical University, Chongqing, China; ^2^Chongqing City Key Lab of Translational Medical Research in Cognitive Development and Learning and Memory Disorders and Ministry of Education Key Lab of Child Development and Disorders, Children’s Hospital of Chongqing Medical University, Chongqing, China; ^3^Department of Clinical Laboratory, The Affiliated Hospital of KMUST, Medical School, Kunming University of Science and Technology, Kunming, China; ^4^Department of Anesthesiology, Children’s Hospital of Chongqing Medical University, Chongqing, China; ^5^Townsend Family Laboratories, Department of Psychiatry, The University of British Columbia, Vancouver, BC, Canada

**Keywords:** *Xenopus*, neural crest, ketamine, Notch, Zic5

## Abstract

Ketamine is a potent dissociative anesthetic and the most commonly used illicit drug. Many addicts are women at childbearing age. Although ketamine has been extensively studied as a clinical anesthetic, its effects on embryonic development are poorly understood. Here, we applied the *Xenopus* model to study the effects of ketamine on development. We found that exposure to ketamine from pre-gastrulation (stage 7) to early neural plate (stage 13.5) resulted in disruption of neural crest (NC) derivatives. Ketamine exposure did not affect mesoderm development as indicated by the normal expression of Chordin, Xbra, Wnt8, and Fgf8. However, ketamine treatment significantly inhibited Zic5 and Slug expression at early neural plate stage. Overexpression of Zic5 rescued ketamine-induced Slug inhibition, suggesting the blockage of NC induction was mediated by Zic5. Furthermore, we found Notch signaling was altered by ketamine. Ketamine inhibited the expression of Notch targeted genes including Hes5.2a, Hes5.2b, and ESR1 and ketamine-treated embryos exhibited Notch-deficient somite phenotypes. A 15 bp core binding element upstream of Zic5 was induced by Notch signaling and caused transcriptional activation. These results demonstrated that Zic5 works as a downstream target gene of Notch signaling in *Xenopus* NC induction. Our study provides a novel teratogenic mechanism whereby ketamine disrupts NC induction via targeting a Notch-Zic5 signaling pathway.

## Introduction

Neural crest (NC), the vertebrate-specific pluripotent cell population, derives from the border region between the epidermis and the neural plate. NC gives rise to numerous derivatives including cranial cartilages, neurons and glia of peripheral nervous system, melanocytes, and heart outflow tract (Shi et al., 2014). Abnormal development of NC derivatives results in neurocristopathies, leading to many human congenital disorders such as Hirschsprung disease, Treacher Collins syndrome, Waardenburg-Shah syndrome, DiGeorge syndrome, CHARGE syndrome, neuroblastoma, and melanoma (Bolande, 1997; Takahashi et al., 2013; Monsoro-Burq, 2015).

The development of NC undergoes induction, specification, migration, and differentiation, which is tightly regulated by a well-orchestrated gene regulatory network (GRN). After germ layer separation, a set of transcription factors including Pax3, Zic1, Zic5, Msx1, Hairy2, Ap2α, and c-Myc are expressed at the border region between the neural plate and the epidermis. Such expression is induced by morphogens like Wnt, BMP, FGF, Notch and retinoic acid in a gradient sensitive manner. These border determination transcription factors further trigger the expression of a group of genes including Slug, FoxD3, Snail, Sox9, and Twist in the emerging NC cells, thereby accomplishing NC induction (Steventon et al., 2005; Milet and Monsoro-Burq, 2012; Shyamala et al., 2015). After induction, the NC cells undergo epithelial to mesenchymal transition (EMT) and migrate to their destination in a contact inhibition of locomotion manner (Theveneau and Mayor, 2012). Terminal differentiation of NC cells is extensively cross-regulated by different networks.

The Notch signaling pathway plays vital roles in early embryonic development (Artavanis-Tsakonas et al., 1995, 1999). Following ligand binding, the Notch receptor is cleaved continuously by an ADAM (Brou et al., 2000; Mumm et al., 2000; Dyczynska et al., 2007; Bozkulak and Weinmaster, 2009) and γ-secretase complex (De Strooper et al., 1999; Song et al., 1999; Zhang et al., 2000) and releases the active N-terminal intracellular domain (NICD). The NICD then translocates to nucleus and interacts with CSL family DNA binding proteins to activate the transcription of downstream target genes (Schroeter et al., 1998; Struhl and Adachi, 1998). Activation of the Notch signaling pathway by binding to adjacent cells facilitates boundary determination. During NC induction, Notch and its ligands are expressed in the prospective anterior NC territory, where Notch coordinates with the BMP signal to determine the NC border region. In somitogenesis, the expressed protein amount of the NICD in nucleus forms a cyclical clock manner through degradation and thereby establish somite boundary formation (Dale et al., 2003).

Notch signaling pathway has been shown to play a role in NC development in different animal models. Disturbance of Notch signaling in mice leads to craniofacial structure abnormity, cardiac outflow deficiency, and decreased enteric neurons (Okamura and Saga, 2008; Mead and Yutzey, 2012). Notch signals refine the neural plate border region through negative regulation of PRDM1α in zebrafish (Hernandez-Lagunas et al., 2011). Studies using chick and *Xenopus* models demonstrate a similar BMP-regulating mechanism of Notch pathways in NC development (Endo et al., 2002). Hairy family genes required for NC induction are down-regulated by BMP-Smad signaling and positively regulated by the Notch/Delta-Su(h) pathway (Vega-Lopez et al., 2015). Another report suggests a role for Tsk in balancing Notch and BMP pathways via directly binding to BMP and the extracellular domain of Delta1 during neural plate border determination in *Xenopus* (Kuriyama et al., 2006).

Ketamine has been commonly used as anesthetic, analgesic, or sedative, and has recently been shown to be a promising acute anti-depressant (Ionescu et al., 2015; Kirby, 2015; Reardon, 2015). As a dissociative anesthetic, ketamine users can develop cravings for the drug and become addicted. Ketamine is now one of the most popular and most abused recreational illicit drugs in the world. A large number of the drug users are women at childbearing ages. The impact of illicit ketamine use on embryonic development during pregnancy could be detrimental. Previous studies using zebrafish, *Xenopus* and rat models showed its disruptive effects on motor-neuron development (Kanungo et al., 2013), primary germ cell layers specification (Akeju et al., 2014), cardiac morphogenesis (Guo et al., 2016), neural genesis and survival (Dong and Anand, 2013; Dong et al., 2014). More recently, a report suggested craniofacial and trunk phenotypes upon embryonic ketamine exposure in Zebrafish (Felix et al., 2017). Furthermore, ketamine has been shown to act as teratogen in a NMDA-independent ways (Felix et al., 2014). Although ketamine’s roles as NMDA antagonist have been extensively studied, the effect on early embryonic development and its underlying mechanisms remain elusive.

In this study, we report that ketamine exposure affected NC development at a very early stage, equivalent to the first month of human pregnancy. Ketamine down-regulates Zic5 expression in the presumptive neural plate border region and thereby disrupting NC induction. Our data indicate that ketamine disturbs Notch signaling, and that Zic5 works downstream of Notch during *Xenopus* NC developmental stages. Our data suggest a completely new teratogenic mechanism whereby ketamine disrupts NC induction via targeting a NOTCH-Zic5 signaling pathway.

## Materials and Methods

### Microinjection and *in Situ* Hybridization

This study was carried out in accordance with the recommendations of the animal study guideline of the Animal Experimentation Ethical Committee of Chongqing Medical University. The protocol was approved by the Animal Experimentation Ethical Committee of Chongqing Medical University in Chongqing, China. Embryo *in vitro* fertilization, culture, whole mount *in situ* hybridization, mRNA preparation and microinjection were carried out as previously described (Shi et al., 2009, 2014). The probes for whole-mount *in situ* hybridization including Chordin, Xbra, Wnt8, Fgf8, Msx1,Pax3, Zic1, Zic5, Slug, Six1, MyoD were applied as described (Smith et al., 1991; Sasai et al., 1994; Nakata et al., 2000; Li et al., 2006; Guemar et al., 2007; Zhang et al., 2014). For enteric neuron labeling, intestines were first manually dissected out from stage 40 embryos which were freshly fixed with 4% paraformaldehyde. Then the enteric neurons were stained through *in situ* hybridization with *N-tubulin* probe (Oschwald et al., 1991).

### Cranial Cartilage Staining, Immunohistochemistry, and RT-PCR

The cranial cartilage staining was performed as previously described (Shi et al., 2014). For immunohistochemistry, embryos were collected at stage 40 and fixed with paraformaldehyde. The frozen samples were sectioned in 10 μm thickness, and stained with β-tubulin III (Sigma T8660). Total RNA samples were extracted with Trizol kit (Tiangen) and reversely transcribed with Fermentas RevertAid First Strand cDNA synthesis kit (Thermoscientific 1622). The RT-PCR primers for *Fgf8*, *Wnt8, Dlx3, Dlx5, AP2α, Pax3, Msx1, Zic1, Zic5, Slug, Hes5.2a*, *Hes5.2b*, *ESR1*, and *Histone4 (H4)* were listed in **Table 1**.

**Table 1:** RT-PCR primers used in this study.

Gene	Primers (forward and reverse)	Cycles	Annealing temperatures (°C)	Extension time (s)	Reference
Fgf8	5′-TGCGGAGACTGGTTACTACATCTG-3′	28	55	30	Monsoro-Burq et al., 2003
	5′-TTCTGTGGTGTGGTGTCCCTTTGG-3′				
Wnt8	5′-GACAAGATGCCAGAGCCCTAA-3′	28	55	30	Zhang et al., 2014
	5′-TAAGTTCAGACCCGGCCACA-3′				
Dlx3	5′-TCGGCCGTTTGTCCATTACA-3′	26	55	30	Zhang et al., 2014
	5′-GGTTTCGGGCTCTTCCTTCA-3′				
Dlx5	5′-ATTCTCCCCAGTCTCCAGTG-3′	26	55	30	This work
	5′-GATAGTGTCCCCAGTTGCGC-3′				
AP2α	5′-GGACCTGCCTTTACATCCATACC-3′	28	55	30	de Croze et al., 2011
	5′-CCTCCGTTTTTAGATTTTGCCC-3′				
Pax3	5′-TCTCACTCTCTCTTTACAGGGGGAC-3′	29	55	30	Monsoro-Burq et al., 2005
	5′-TCTTGTGCCTTATGTGGTTGGG-3′				
Msx1	5′-ACTGGTGTGAAGCCGTCCCT-3′	29	55	30	Su et al., 1991
	5′-TTCTCTCGGGACTCTCAGGC-3′				
Zic1	5′-ATGAAGGTCCACGACGAAGCATC-3′	30	55	30	Mizuseki et al., 1998
	5′-CGTGCTGTGATTGGACGTGT-3′				
Zic5	5′-AGAGAGGACTATACGCTAAC-3′	27	55	30	Nakata et al., 2000
	5′-GGTACATGAGAGCAGAGAAC-3′				
Slug	5′-TCCCGCCACTGAAAATGCCACGATC-3′	28	55	30	Mizuseki et al., 1998
	5′-CCGTCCTAAAGATGAAGGGTATTCCTG-3′				
Hes5.2a	5′-GGCATTGGTAGAAGCAGTC-3′	28	55	30	This work
	5′-CCAGTTTAACCTTGGGTGTC-3′				
Hes5.2b	5′-AGCAGAAGCCAAGCTAATC-3′	28	55	30	This work
	5′-AGGACCATAACCGAACAAG-3′				
ESR1	5′-ACAAGCAGGAACCCAATGTCA-3′	28	55	30	Kinoshita et al., 2011
	5′-GCCAGAGCTGATTGTTTGGAG-3′				
H4	5′-CGGGATAACATTCAGGGTATCACT-3′	24	55	30	Kinoshita et al., 2011
	5′-ATCCATGGCGGTAACTGTCTTCCT-3′				


### Western Blot Analysis

T-Leukemia cell lines Jurkat were cultured with 2 mM ketamine for 10 h and collected for WB. The Antibodies used were Notch-1 antibody (Cell Signaling 3608, 1:1000), GAPDH (Enogene E12-052-4, 1:5000). For Notch ubiquitin assay, 5 μM MG132 (Sigma) was added with or without ketamine into the media, Jurkat cells were then lysed and precipitated with Notch-1 antibody (1:50) and then bound with protein A/G (Santa Cruz sc-2003). Anti-ubiquitin (Santa Cruz sc-8017) antibody were applied to detect ubiquitinated proteins.

### Luciferase Reporter Assays

Different fragments (100 bp–4 kb) upstream of *Xenopus tropicalis Zic5* gene were cloned and linked with *Xho*I and *Mlu*I restrictive site into pGL3-basic to generate the firefly reporter constructs. The primers for different lengths of *X. tropicalis Zic5* upstream regulatory sequences are: reverse primer *Xho*I: 5′-ccctcgagtgtctgcctcccaactct; -4042 forward: 5′-cgacgcgttgagatggcgagtaggct; -1950 forward *Mlu*I: 5′-cgacgcgttccttattagtgtatata; -532 forward: 5′-cgacgcgttgcacaactataggtctatt; -286 forward: 5′-cgacgcgtctccaaactttctacaagtg; -200 forward: 5′-cgacgcgtcagccagccaatcagaaaag; -200m forward: 5′-cgacgcgtttctaattaaggattgaaaagcgggcctcc; -186 forward: 5′-cgacgcgtgaaaagcgggcctcctgcc. To mutant canonical CSL binding site, mutant kit (Takara R401) as well as template construct pXzic5 -4042/-29 was applied according to the manufacture with primers: for 3950 site forward 5′-atgtattgcttcgaccttggcattttgg, reverse 5′-gtttctacattttccaaagttgtgcaaag; for 65 site forward 5′-ggacagacctacggaaggaataagc, reverse 5′-ctccagcgattggcagaaagcg.

To measure the reporter activity in embryos, 12.5 pg constructed firefly plasmid, 2.5 pg renilla construct and 500 pg NICD mRNA or 500 pg LacZ mRNA (for control) were co-injected into both side of the dorsal blastomeres at the 4-cell stage. The embryos were then harvested at the neural plate stage (stage 15), divided into three groups (>10 embryos each group), lysed and analyzed using Dual-Luciferase Reporter Assay System (Promega).

### Electrophoretic Mobility Shift Assay (EMSA)

SHSY-5Y cell line was transfected with pcDNA4-NICD or pcDNA empty vector. Nuclear protein was extracted. EMSA was performed as described previously with minor adjustment (Wang et al., 2011). Briefly, 20 μg protein was incubated with IRDye700-labeled binding oligo (5′-gcctgacagccagccaatca) or mutant oligo (5′-gactaacaaccatccaaaca) respectively, and the gels were analyzed using an Odyssey system (LI-COR Biosciences). Unlabeled wild-type and mutant oligonucleotides at X100 molar excess were applied for the competition assay.

## Results

### Embryonic Ketamine Exposure Results in Neurocristopathies

**FIGURE 1:**
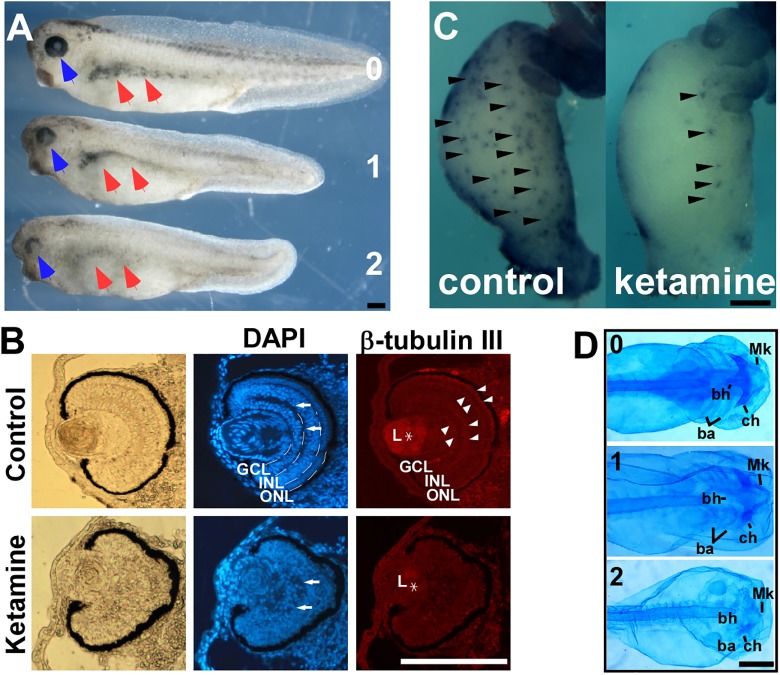
Ketamine exposure results in neurocristopathies. Upon ketamine exposure from stage 7 to stage 13.5. **(A)** Embryos exhibit shortened axis, faded trunk pigment (red arrowhead), and deficient eyes (blue arrowhead) at tailbud stage and severity of phenotypes are in a dose-dependent manner. **(B)** Sections of eyes in ketamine-treated embryos revealed a more rounding shape pigment, disorganized arrangement of cells (DAPI staining), reduced expression of β-tubulin III compared with the control group. Ketamine exposure also causes embryos to develop a mega-colon like phenotype **(C)**, and cranial cartilage atrophy **(D)**. GCL, ganglionic cell layer; INL, inner nuclear layer; ONL, outer nuclear layer; L, lens; Mk, Meckel’s cartilage; ch, ceratohyal cartilage; bh, basihyal cartilage; ba, branchial cartilage. Numbers in **(A,D)** indicate ketamine exposure concentration (mM) to embryos. Scale bar: **(A–C)** 100 μm, **(D)** 1 mm.

Previous data indicates that 2 mM ketamine applied in culture yields approximately 0.4% accumulation, i.e., 8 μM inside embryo (Cuevas et al., 2013), which is comparable to the anesthetic concentration in human plasma (Dong et al., 2014). 2 mM or similar culture concentration was commonly chosen to evaluate the embryonic ketamine exposure in most studies (Kanungo et al., 2013; Felix et al., 2014; Lantz-McPeak et al., 2015; Robinson et al., 2015). To examine ketamine’s effects on early embryonic development, we exposed *Xenopus* embryos to 2 mM ketamine-MBS culture media from pre-gastrulation (stage 7) to early neural plate (stage 13.5), and then cultured in MBS media until harvest time. Upon ketamine incubation, pigment cells in trunk diminished and eyes exhibited developmental abnormities (**Figure 1A**). Ocular pericytes located in retina ganglionic cell layer (GCL) and inner nuclear layer (INL) are derived from the NC (Trost et al., 2013). Further analysis showed disorganization of GCL, INL, and outer nuclear layer (ONL) (**Figure 1B** DAPI stain, white arrow) and loss of β-tubulin III expression in cell layer boundary (**Figure 1B**, white arrowhead) and lens (**Figure 1B**, asterisk) expression, indicating abnormal differentiation of lens and retina. At the tailbud stage, ketamine caused enlargement of intestinal system combining loss of enteric nervous cells (**Figure 1C**, black arrowhead), a mega-colon like (Hirschsrpung disease) phenotype (**Figure 1C**) and marked reduction or even complete loss of all the cranial cartilage elements including Meckel’s (Mk), ceratohyal (ch), basihyal (bh), and branchial (ba) cartilages as demonstrated by alcian blue staining (**Figure 1D**). These data clearly indicate that ketamine impairs NC development, causing neurocristopathies.

### Ketamine Blocks NC Induction Independent of Mesoderm Genesis

**FIGURE 2:**
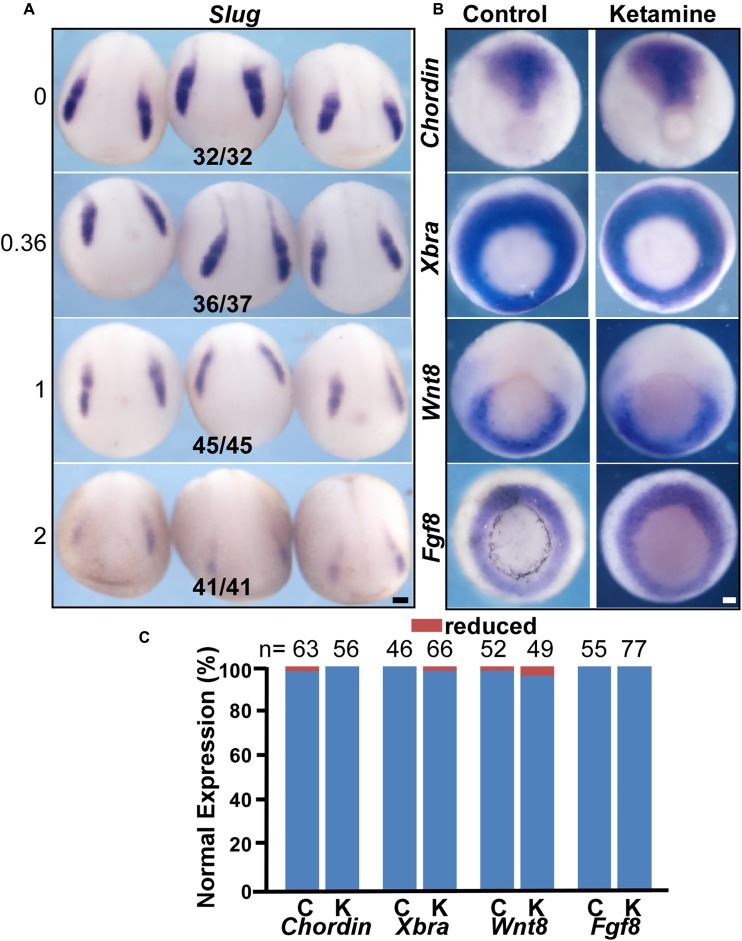
Ketamine blocks NC induction independent of mesoderm genesis. **(A)** The expression of the NC marker gene *Slug* was inhibited by ketamine in a dose-dependent manner. The numbers of embryos showing similar changes in gene expression and total embryos in each concentration group are indicated. **(B)** Early mesoderm development is not affected upon ketamine exposure. There were no significant differences in the expression pattern of mesoderm marker genes *chordin*, *Xbra*, *Wnt8*, and *Fgf8* between control and ketamine-treated group prior to NC induction. **(C)** Quantitative analysis of gene expression patterns between control and ketamine group. Scale bar: 100 μm.

Induction is the first event in NC genesis. To investigate the effect of ketamine on NC induction, we examined the expression pattern of the NC specification gene *Slug*. The criteria for judging the expression pattern are based on two aspects, expression area and intensity. Compared to control, decreased expression area or signal intensity was determined as ‘reduced expression level.’ At the early neural plate stage (stage 14), when the NC has been induced, control embryos express a normal *Slug* expression pattern (in 32/32 embryos) (**Figure 2A**). However, ketamine at a 0.36 mM culture concentration showed reduced *Slug* expression (36/37). The inhibition of *Slug* expression increases in a dose dependent manner. At a 2 mM ketamine concentration, *Slug* expression was severely reduced (41/41) (**Figure 2A**). Morphogens from the mesoderm are critical for inducing the NC (Klymkowsky et al., 2010). Thus, we examined mesoderm development by *in situ* hybridization assay with mesoderm markers on the ketamine-incubated and control embryos prior to NC induction (stage 11.5) (**Figure 2B**). There was no significant difference between ketamine and control groups in the expression pattern of four mesoderm maker genes including *Chordin* (100% normal expression for ketamine, *n* = 56; and 98.41% normal expression for control, *n* = 63), *Xbra* (98.48% normal expression for ketamine, *n* = 66; and 100% normal expression for control, *n* = 46), *Wnt8* (95.92% normal expression for ketamine *n* = 49; and 98.08% normal expression for control, *n* = 52), and *Fgf8* (100% normal expression for ketamine, *n* = 77; and 100% normal expression for control, *n* = 55) (**Figure 2C**). These results suggest that ketamine does not affect mesoderm at the beginning of NC induction. These data indicate that ketamine blocks NC induction independent of mesoderm genesis.

### Ketamine Disrupts NC Induction by Inhibiting the Expression of *Zic5*

**FIGURE 3:**
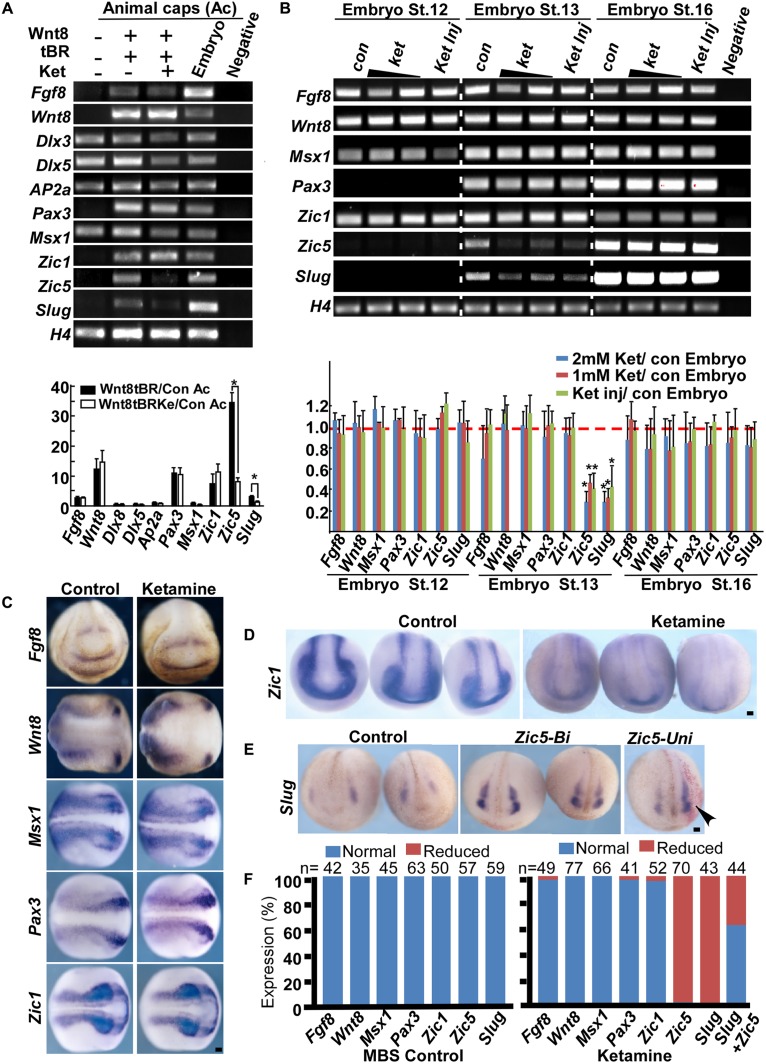
Ketamine targets *Zic5* in NC induction. **(A)** In animal caps, Wnt8 (500 pg) and tBR (500 pg) induce expression of NC markers (lanes 1, 2). Upon ketamine exposure, the expression of *Zic5* and *Slug* were inhibited (lanes 2, 3). The induced expression of *Zic5* and *Slug* were reduced by ketamine treatment in animal cap. Gene expressions were normalized with H4. Data are shown as folds over the control animal caps. 15 animal caps were isolated for each group for single time experiments. The number represents Mean ± SEM, *N* = 3, ^∗^*P* < 0.05 by ANOVA. **(B)** In whole embryos, either ketamine exposure or injection (0.8 nmol/embryo) blocked *Zic5* and *Slug* expression at stage 13 (when the NC is being induced) but not stage16 (at the onset of NC migration). Ketamine treatment did not affect most of the border genes from stage 12 to stage 16, but significantly blocked *Zic5* and NC gene *Slug* at stage 13. For a single time experiment, 10 embryos were collected for each group for RT-PCR. Data are shown as folds over the control embryos. The number represents Mean ± SEM. *N* = 3, ^∗^*P* < 0.05 by ANOVA. **(C)** *In situ* hybridization suggests genes involved in NC induction, including *Fgf8*, *Wnt8*, *Msx1*, *Pax3*, and *Zic1* were not affected by ketamine exposure. **(D)** Ketamine inhibited *Zic5* expression comparing with MBS cultured control group. **(E)** The inhibition of *Slug* expression by ketamine was rescued by injection of *Zic5* mRNA (500 pg) in both sides or unilaterally. Arrowhead indicate the unilaterally injected side. **(F)** Quantitative analysis of *in situ* hybridization results, left: MBS control group, right: ketamine exposure group. ^∗^*P* < 0.05 with *t*-test. Con, control; Ket, ketamine; Ket Inj, ketamine injection; St., stage; Zic5-Bi, zic5 bilateral injection; Zic5-Uni, zic5 unilateral injection. Scale bar: 100 μm.

Neural crest induction requires high Wnt and intermediate BMP signaling input (Steventon et al., 2009). At the onset of gastrulation, animal cap, a pluripotent cell group located in at the animal pole of *Xenopus* embryos, can develop into almost any cell type given the proper signaling stimulation *in vitro*. In *Xenopus* animal caps, co-injection of Wnt8 and BMP4 truncated receptor (tBR) mimics endogenous NC stimulation and results in the expression of NC genes (Zhang et al., 2014). To determine the targeting molecules mediating ketamine’s effect on NC induction, we first examined gene expression in the wnt8-tBR co-injected animal cap system. The default of animal cap cells is an epidermis fate, characterized by a high BMP signaling niche (Linker et al., 2009; Montagner et al., 2016). BMP target genes Msx1 (Tribulo et al., 2003), Dlx3, Dlx5 (Luo et al., 2001; McLarren et al., 2003) and AP2α (Luo et al., 2002, 2003) are involved in neural plate border determination and are normally expressed in control animal caps. Co-injection of 500 pg *Wnt8* mRNA and 500 pg *tBR* mRNA successfully triggers animal cap cells to express the secreted genes *Fgf8*, *Wnt8*, and neural border genes including *Zic1*, *Pax3*, *Zic5* as well as NC specific genes *Slug* (**Figure 3A**). In the presence of ketamine, Wnt8-tBR induced animal caps markedly reduces the expression of *Zic5* and *Slug* (**Figure 3A**). *Zic5* is expressed in the NC area and plays an indispensable role in the induction process (Nakata et al., 2000). Since embryos undergo NC induction in the late gastrula (approximate at stage 12.5), we examined the NC markers at this critical time with a 1–2 mM ketamine exposure or 0.8 nmol direct injection. At stage 12 the pre-induction state, neither control nor ketamine-treated embryos expressed *Slug*. At stage 13, all of the NC regulatory network genes were expressed in control embryos, whereas *Zic5* and *Slug* transcripts were reduced in embryos cultured in the ketamine-containing media or directly injected with ketamine. However, the *Zic5* and *Slug* expression levels from whole embryos returned to normal at the beginning of NC migration (stage 16). The recovery NC gene expression may be due to the feedback of complicated GRNs (**Figure 3B**). Consistent with RT-PCR data from animal caps and whole embryos, *in situ* hybridization results showed that ketamine treatment did not affect most NC induction-related genes including *Fgf8* (97.96% normal expression, *n* = 49), *Wnt8* (100% normal expression, *n* = 77), *Msx1* (100% normal expression, *n* = 66), *Pax3* (97.56% normal expression, *n* = 41), and *Zic1* (96.15% normal expression, *n* = 52) (**Figures 3C,F**). However, the expression of *Zic5* and the induction marker gene *Slug* were markedly inhibited by ketamine, with 97% (*n* = 70,) and 100% (*n* = 43) of embryos having inhibited expression respectively (**Figures 3D,F**). Finally, exogenous injection of *Zic5* mRNA at two-cell stage significantly rescued the ketamine-induced *Slug* inhibition (63.64% of normal expression, *n* = 44) (**Figures 3E,F**). Our data suggest that ketamine disrupts NC induction by inhibiting Zic5 expression.

### Targeting *Zic5* by Notch Signaling in NC Induction

**FIGURE 4:**
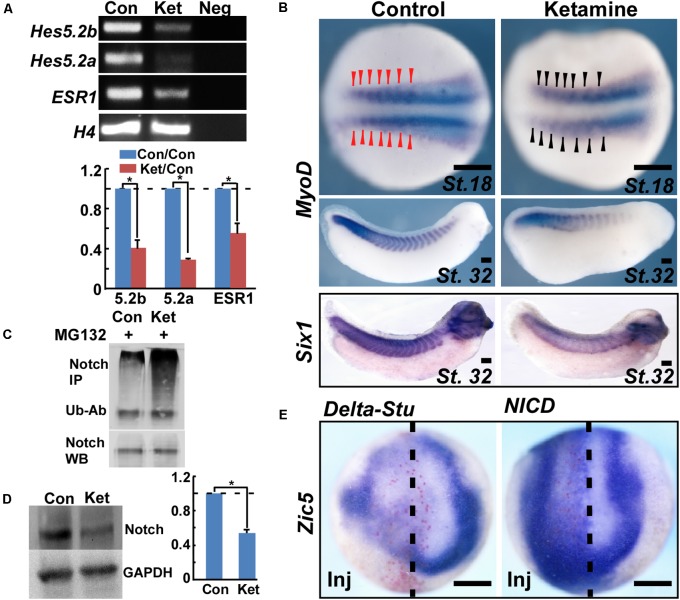
Ketamine inhibits *Zic5* through the Notch signaling pathway. **(A)** During NC induction, ketamine exposure down-regulated expression of Notch targeted genes including *Hes5.2a*, *Hes5.2b*, and *ESR1.* The number represents Mean ± SEM, *N* = 3, ^∗^*P* < 0.05 by Student’s *t*-test. **(B)** In the late neurula, *MyoD* transcription in early somite primordium became a little bit thin and slightly reduced the signal upon ketamine exposure. At tailbud stage, ketamine led to fewer somites and a shortened body axis. The somites are labeled with *in situ* hybridization of *MyoD*, and *Six1*. **(C)** Ketamine exposure increased the amount of ubiquitinated Notch protein in Jurkat cells. Upper part: ubiquitinated Notch proteins were immuno-precipitated (IP) with Notch-1 antibody, followed by anti-ubiquitin antibody (Ub-Ab) western blot staining. Lower part: Notch-1 loading control. **(D)** In Jurkat cells, ketamine exposure for 10 h reduced Notch protein level. The number represents Mean SEM, *N* = 3, *P* < 0.05 by Student’s *t*-test. **(E)** During NC induction, inhibiting Notch signaling by microinjection of 1 ng Delta-stu mRNA at 4-cell stage blocked Zic5 expression. Activation of Notch signaling by microinjection of 800 pg NICD mRNA into one dorsal cell at 4-cell stage induced ectopic Zic5 expression. Scale bar: 100 μm.

To investigate the upstream signaling pathways mediating ketamine-induced *Zic5* inhibition during NC induction, we screened the most common NC induction morphogens including Wnt, Fgf, and Notch. Previous work showed that prospective paraxial mesoderm or *Fgf8* induces *Zic5* expression (Monsoro-Burq et al., 2003). Our results show that *Fgf8* and its downstream NC inducing gene *Msx1* (Monsoro-Burq et al., 2005) were normally expressed and not affected by ketamine treatment (**Figure 3**). However, ketamine exposure markedly reduced the expression of Notch target genes *Hes5.2a*, *Hes5.2b*, and *ESR1* (**Figure 4A**). Somite labeling with *MyoD* and *Six1* transcripts showed slightly reduced signal (**Figure 4B**) and a shortened embryonic body axis (**Figure 1A**) that mirrors phenotypes seen in Notch-deficient embryos (Jen et al., 1997). To analyze ketamine’s effect on Notch signaling, the human T-cell leukemia line Jurkat was used. 2 mM Ketamine exposure for 10 h increased the amount of ubiquitinated Notch (**Figure 4C**) and promoted Notch protein degradation (**Figure 4D**). To ask whether *Zic5* functions as a downstream target gene of Notch signaling, we assayed *Zic5*’s response to high and low Notch environment during NC induction in *Xenopus* embryos. Blocking Notch pathway with microinjection of *Delta-stu* mRNA (Revinski et al., 2010) at the 4-cell stage markedly inhibited *Zic5* expression (**Figure 4E**). Activation of Notch signaling by injection of *NICD* (Fryer et al., 2002) induced *Zic5* ectopic expression in whole neural plate and NC area (**Figure 4E**).

### Identification of a 15 bp *cis*-Acting Notch-Response Element in the *Zic5* Upstream Regulatory Region

**FIGURE 5:**
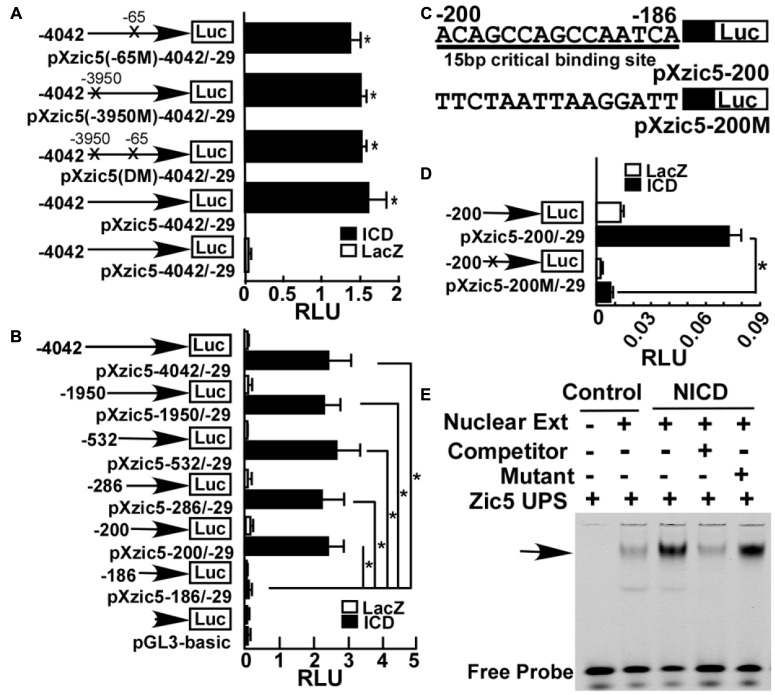
Identification of a novel NRE in *Xenopus Zic5*. **(A)** A ∼4 kb region (-4042 to -29) upstream of *Zic5* contains a *Zic*5 promoter. Mutations of two putative canonical CSL binding sites within this 4 kb region did not affect notch’s activation of promoter activity. X’s represent the mutated site. **(B)** Deletion assay of Zic5 upstream regulatory sequence identified a 15 bp NRE locating between -200 and -186 bp in response to NICD activation. *P*-values relative to -186/-29 fragment are -200/-29 (*P* = 0.004); -286/-29 (*P* = 0.015); -533/-29 (*P* = 0.013); -1950/-29 (*P* = 0.004); -4042/-29 (*P* = 0.014). **(C)** Diagram of the constructs containing 15 bp CSL sequence and mutations in the promoter constructs. pXZic5-200 represents the wild-type and pXZic5-200M represents the 15 bp CBS mutant plasmid. **(D)** Mutation of the 15 bp CBS (pXZic5-200M) abolished the activation triggered by NICD in the reporter assay; *P* = 0.0017. **(E)** EMSA assay; NICD expression increased protein binding to the 15 bp NRE (see section “Materials and Methods”). The number represents Mean ± SEM, *N* = 3, ^∗^*P* < 0.05 with the *post hoc* Newman–Keuls test. RLU, relative luminant unit; Nuclear Ext, nuclear extraction; Zic5 UPS, fluorescent-labeling zic5 upstream Notch response element.

To investigate the molecular mechanism underlying Notch’s effect on *Zic5*, we first examined whether the *Zic5* gene contains Notch response element (NRE) in its upstream regulatory region. A 4013 bp containing sequence, between -4042 and -29 bp upstream of the translation start codon (designated as +1), for the *X. tropicalis Zic5* gene was cloned into pGL3-basic to generate a *firefly luciferase* gene reporter construct pXzic5-4042/-29. Sanger sequencing confirmed the insertion of -4042 to -29 as identical to 117367902–117371914 of chromosome 2 (Xenbase/*X. tropicalis* Gbrowse 9.0). This 4 kb sequence contains two putative canonical Notch signaling CSL binding elements (GTGGGAA) in -65 to -58 (chromosome 2, 11731879–11731885) and in -3950 to -3944 (chromosome2, 11731971–11731977) upstream of *Zic5*. To check the promoter activity and the involvement of these two CSL *cis*-acting binding elements, pXzic5 (-65M)-4042/-29 carrying the -65 binding site mutation to CCTACGG, pXzic5 (-3950M)-4042/-29 carrying the -3950 binding site mutation to CCTACGG, and pXzic5 (DM) -4042/-29 carrying both binding site mutations to CCTACGG were generated for reporter assays (**Figure 5A**). *NICD* mRNA or *LacZ* mRNA (mock stimuli), and pXZIC5-4042/-29 or relative 4 kb insertion containing CSL mutant firefly constructs were co-injected into both dorsal blastomeres (that target the neural plate and NC area) at 4-cell stage. A *Renilla luciferase* construct was also co-injected into embryos as the normalization control. Upon *NICD* stimulation, pXzic5-4042/-29 (1.59 ± 0.23 RLU), pXzic5 (DM)-4042/-29 (1.51 ± 0.06 RLU), pXzic5 (-3950M)-42/-29 (1.49 ± 0.07 RLU) and pXzic5 (-65M)-42/-29 (1.37 ± 0.13 RLU) reveal significant high luciferase expression level (*P* < 0.05) compared with negative control (LacZ) stimulated pXzic5-4042/-29 (0.05 ± 0.01 RLU) group in *Xenopus* Embryos. Transcriptional activity exhibited no statistical difference among the 4 kb wild-type and the putative CSL-binding site mutant constructs (**Figure 5A**). Our data suggest that there is functional NRE within region -4042 to -29, however, the two putative canonical CSL binding sites did not account for Notch-activated Zic5 transcriptional activation.

To identify the NRE, a series of deletion plasmids, covering various regions of this 4 kb fragment were cloned. **Figure 5B** showed that NICD significantly activated the luciferase report plasmids containing -4042 bp (2.49 ± 0.62 RLU), -1950 bp (2.36 ± 0.45 RLU), -532 bp (2.73 ± 0.063 RLU), -286 bp (2.30 ± 0.62 RLU), and -200 bp (2.48 ± 0.42 RLU) upstream of Zic5 ORF with similar activation. A further deletion of 15 bp lead to a sharply reduced luciferase activity in pXzic5-186/-29 (0.187 ± 0.099RLU) (*P* < 0.05) (**Figure 5B**). Therefore, the 15 bp sequence (5′-ACAGCCAGCCAATCA) located between -200 and -186 bp upstream of *Zic5* was identified to contain a functional NRE. To confirm the results, we generated a new plasmid pXzic5-200M containing mutations in the NRE (**Figure 5C**). The Zic5 upstream 200 bp reporter plasmid containing the 15 bp NRE was strongly activated by NICD (0.073 ± 0.0069 RLU), and the mutations in the 15 bp sequence to disrupt the NRE (0.007 ± 0.0009 RLU) abolished the activation of Zic5 by NICD (**Figure 5D**).

Electrophoretic mobility shift assay (EMSA) was performed to further examine whether Notch signaling activation promotes the binding of the transcriptional factors to the 15 bp NRE. Nuclear proteins with or without NICD-transfected were extracted from SHSY5Y cells. NICD-transfection significantly increased the amount of the protein-NRE DNA binding complex (**Figure 5E**). The competitor probe containing the same non-fluorescently labeled 15 bp sequence reversed the fluorescent binding signaling to background level, and a mutant probe had little effect to compete with the binding (**Figure 5E**). These results unequivocally demonstrate that Notch activates Zic5 expression via the newly identified 15 bp NRE.

## Discussion

Ketamine is a dissociative anesthetic and the most popular club drug abused by youths for recreational purposes (Lee et al., 2015). Lately ketamine has attracted extensive attention for its quick and significant alleviation of depression (DiazGranados et al., 2010; Li et al., 2011). Ketamine usage grows steadily. Young people, including those at childbearing age, are a major addict-prone population. In human, NC induction happens within the first month of pregnancy. At this early point in the pregnancy, most women are not aware that they are pregnant until the next regular menstrual cycle. While ketamine exposure has been shown to affect cell survival (Brambrink et al., 2012; Bai et al., 2013), germ layer specification (Akeju et al., 2014), neurogenesis (Cuevas et al., 2013; Kanungo et al., 2013; Dong et al., 2014), cardiac morphogenesis (Guo et al., 2016), and craniofacial and trunk genesis (Felix et al., 2017), the effect of ketamine on early embryonic development is poorly understood.

In this study, we discovered that early embryonic ketamine exposure specifically blocks NC induction via targeting Zic5. This effect on the NC is not due to the disruption of the mesoderm. Previous studies suggested the vital roles of Zic5 in NC genesis in mouse and *Xenopus* (Nakata et al., 2000; Inoue et al., 2004). Zic5 also works downstream of Wnt pathways and regulates tectum cell proliferation in zebrafish (Nyholm et al., 2007). However, the upstream signals regulating Zic5 expression in NC induction were not yet known. Our work revealed that the expression of Zic5 is controlled by Notch signaling during NC induction. Ketamine inhibited Notch targeted gene expression in *Xenopus* embryos. *Xenopus* embryos exposed to ketamine also exhibited shortened somite-segmentation, a typical Notch signaling deficiency phenotype. The Notch signaling pathway has long been known to participate in NC induction (Glavic et al., 2004; Kuriyama et al., 2006). In the neural-epidermal boundary, Notch balances BMP via activation of Hairy2 expression (Glavic et al., 2004). However other reports showed that Hairy2 is not regulated by Notch, but activates Notch via Stat3 (Nichane et al., 2008a,b). In this study, we demonstrated that Zic5 is a Notch targeted border gene involved in *Xenopus* NC induction. We also identified a 15 bp *cis*-acting element in the 5′ regulatory region of *Xenopus* Zic5 gene that binds to NICD to mediate Notch’s transcriptional activation of Zic5 gene expression. Our study further showed that ketamine enhanced ubiquitination of Notch proteins, and reduced Notch-1 protein level. These data suggest that enhanced degradation of Notch protein by ketamine may partly account for the phenotypes we observed in *Xenopus*, consistent with the similar phenotypes in Zebrafish (Felix et al., 2017). These results suggest a conserved mechanism of ketamine among different spices.

In summary, we discovered that Zic5 works as a downstream target gene of Notch signaling in *Xenopus* NC induction. Our study provides a novel teratogenic mechanism for ketamine whereby it disrupts NC induction by targeting a Notch-Zic5 signaling pathway.

## Author Contributions

YS, JL, and WS conceived and designed the experiments. YS, JL, CC, YXi, YL, and YXu performed the experiments. YS, JL, YXi, YXu, TL, and WS analyzed and contributed reagents/materials/analysis tools. YS, JL, and WS wrote the paper. All authors reviewed the manuscript.

## Conflict of Interest Statement

The authors declare that the research was conducted in the absence of any commercial or financial relationships that could be construed as a potential conflict of interest.
